# The Development and Analysis of Integrated Neuroscience Data

**DOI:** 10.3389/fncom.2016.00011

**Published:** 2016-02-11

**Authors:** Joshua I. Glaser, Konrad P. Kording

**Affiliations:** ^1^Interdepartmental Neuroscience Program, Northwestern UniversityChicago, IL, USA; ^2^Department of Physical Medicine and Rehabilitation, Northwestern University and Rehabilitation Institute of ChicagoChicago, IL, USA; ^3^Department of Physiology, Northwestern UniversityChicago, IL, USA; ^4^Department of Applied Mathematics, Northwestern UniversityChicago, IL, USA

**Keywords:** data integration, technology integration, large-scale data analysis, multimodal, neural data analysis

## Abstract

There is a strong emphasis on developing novel neuroscience technologies, in particular on recording from more neurons. There has thus been increasing discussion about how to analyze the resulting big datasets. What has received less attention is that over the last 30 years, papers in neuroscience have progressively integrated more approaches, such as electrophysiology, anatomy, and genetics. As such, there has been little discussion on how to combine and analyze this multimodal data. Here, we describe the growth of multimodal approaches, and discuss the needed analysis advancements to make sense of this data.

## Evolution of Neuroscience Technologies and Questions

The development of neuroscience technology has been rapidly advancing (Stevenson and Kording, 2011; Insel et al., 2013; Kandel et al., 2013; Marblestone et al., 2013) across many approaches, including those that investigate neural activity (Kording, 2011; Prevedel et al., 2014; Schwarz et al., 2014; Van Horn and Toga, 2014; Vladimirov et al., 2014; Hamel et al., 2015; Lemon et al., 2015), neuroanatomy (Zador et al., 2012; Helmstaedter, 2013; Van Essen, 2013; Oh et al., 2014; Glaser et al., 2015), and gene expression and genetics (Cahoy et al., 2008; Stein et al., 2012; Lee et al., 2014; Medland et al., 2014). Advancing technologies allow us to answer more complex questions. For instance, with single electrodes, researchers could only ask about how individual neurons respond to stimuli and relate to behavior (Hubel and Wiesel, 1959; O’Keefe and Dostrovsky, 1971; Georgopoulos et al., 1982). With the invention of electrode arrays (Maynard et al., 1997; Schwarz et al., 2014; Siegel et al., 2015) and large-scale optical recording techniques (Prevedel et al., 2014; Vladimirov et al., 2014; Hamel et al., 2015), many now ask how neurons interact with each other (Cohen and Kohn, 2011; Stevenson and Kording, 2011; Cunningham and Yu, 2014). Data analysis techniques have been extended to make sense of this growing neural data (e.g., Pfau et al., 2013; Cunningham and Yu, 2014; Freeman et al., 2014; Gao and Ganguli, 2015), which has led to many important insights about the brain.

Along with developing new technologies and increasing the scalability of existing technologies, another way to answer more complex questions is to combine multiple approaches (e.g., using electrophysiology and neuroanatomy together). The brain is a complex system whose function depends on the interplay between countless structures and actions, all spanning different spatial and temporal scales. Combining multiple approaches is critical for understanding how different aspects of the brain relate to each other, e.g., how the morphology of a neuron influences its activity. Moreover, combining multiple approaches is critical for understanding how the brain operates at multiple scales, e.g., how the spikes of individual neurons are related to waves of activity spread across the brain. Data analysis techniques to make sense of this “multimodal” data will be very important going forward.

## Growth of Multimodal Approaches

Multimodal approaches have been used for many years. As a classic example, Hubel and Wiesel (1962) used electrophysiology and anatomy to determine how the functional properties of cells were different across different layers of visual cortex. As a more recent example, researchers have simultaneously used gene expression techniques and tracing (anatomy) techniques to determine what cell types are connected to each other (Sorensen et al., 2015). Similarly, the Allen Brain Institute has been developing an atlas with integrated connectivity, gene expression, and neuroanatomical information (Sunkin et al., 2013). Such approaches allow us to understand how different modalities relate to each other, and how they together lead to brain function.

**Figure 1:**
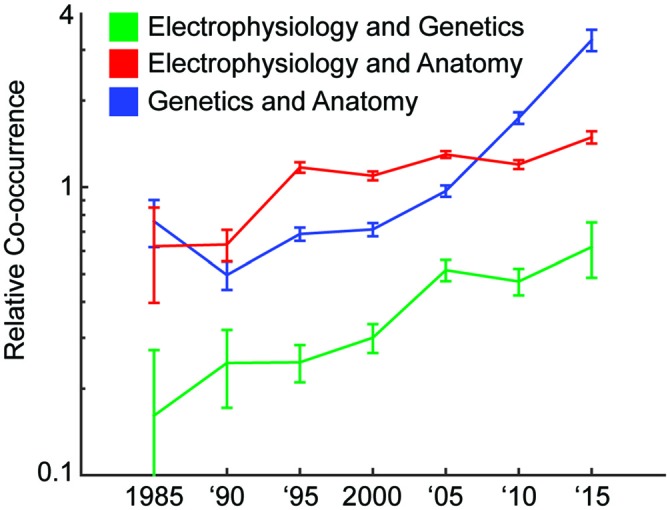
**Increase of multimodal papers over time.** We track how often different modalities of technology are used together over 5 year intervals. We do this by searching the PubMed database for papers with subjects representing the modalities. A relative co-occurrence (*y*-axis; plotted on a log-scale) of one means that occurrence of the two modalities in a paper is independent. A value greater than one means they are more likely to appear together, and a value less than one means they are more likely to appear apart.^1^

Have the amount of studies that combine technologies from multiple experimental modalities (Bock et al., 2011; Hofer et al., 2011; Annese, 2012; Sui et al., 2012; Sunkin et al., 2013; Marblestone et al., 2014; Uludağ and Roebroeck, 2014; Markram et al., 2015; Sorensen et al., 2015) been increasing? We looked in the PubMed database for the number of neuroscience articles that had anatomy, genetics, and/or electrophysiology (one common method of collecting activity) as a subject matter. Over time, the probability of two modalities co-occurring in the same paper increased, for each combination of the three modalities (Figure 1). Interestingly, this increase has occurred at different rates for different combinations of modalities (Figure 1); over the last 30 years, the relative co-occurrence of electrophysiology and anatomy has doubled, and the relative co-occurrences of electrophysiology and genetics, and anatomy and genetics have quadrupled. The integration of approaches is clearly accelerating.

## Gathering Multimodal Data to Understand Neural Activity

For the remainder of the paper, we focus on how multimodal approaches can help us understand neural activity, at the level of neurons (electrophysiology or calcium imaging, as opposed to neuroimaging). Prior to discussing how to analyze this data, it’s important to elaborate upon how this multimodal data can be acquired.

First of all, in many cases, we can gather information about additional modalities with standard activity (electrophysiology or calcium imaging) experiments. Trivially, activity recordings come with approximate neuroanatomy information. That is, we generally know what area of the brain is being recorded from. Additionally, we can get approximate information about cell type (inhibitory vs. excitatory) from electrophysiological waveforms (Mitchell et al., 2007). We can even get approximate estimates of structural connectivity using neural activity (Keshri et al., 2013; Fletcher and Rangan, 2014; Veeriah et al., 2015). Thus, truly multimodal experiments may not always be necessary to gather some forms of multimodal data.

Next, information about multiple modalities can be acquired via more complex experiments. For example, researchers have used calcium imaging followed by electron microscopy in order to determine the relation between neuroanatomy (e.g., connectivity) and neural activity (Bock et al., 2011). As another example, researchers have utilized modern genetic techniques to define cell types via gene expression, and then determine how neural activity differs between those cell types (e.g., Pinto and Dan, 2015). These experiments directly provide rich data from multiple modalities, and are critical for providing a ground truth about how modalities interact.

Lastly, it may be possible to combine information from multiple experiments from different subjects. While we generally cannot match specific neurons across subjects^2^, we can utilize statistical information from previous experiments. For example, we can use information about the likelihood of neurons being connected as a prior in a model that aims to explain neural activity (Rigat et al., 2006; Mishchenko et al., 2011). Additionally, previous information about the relationship between activity and another modality can be used. For example, suppose that cell types can be determined by looking at neural activity in response to varying stimuli (Farrow and Masland, 2011). Future experiments could first determine the cell type based on this previous knowledge, and then see how cell type relates to activity under novel experimental conditions. Utilizing data or knowledge from previous experiments can lead to important multimodal findings.

## Analyzing Multimodal Data

Neural recording is scaling up, and multimodal approaches are increasing. There has been much discussion about how to analyze and build models from large neural activity datasets (Eliasmith and Trujillo, 2014; Gao and Ganguli, 2015; O’Leary et al., 2015). However, there has been little discussion about how additional modalities should be utilized for analyzing and modeling neural activity. These analyses will be crucial for determining how the interplay between different modalities leads to neural function.

One way to analyze this multimodal data is simply to use current analysis methods, and gain additional knowledge by labeling the results based on information about another modality. For example, we generally model the activity of a single neuron based on external factors (e.g., movement or stimuli) and the activity of other neurons (Stevenson et al., 2012; Fernandes et al., 2014; Park et al., 2014). With knowledge from another modality (e.g., cell type), we could first use this same approach. Then, we could look at the results in terms of the cell type to answer questions such as: Do the different cell types respond differently to external factors, and are neurons of certain cell types more likely to be functionally connected? Thus, when modeling the activity of single neurons, standard analysis approaches may be sufficient to answer some questions.

Similarly, it is possible to use current analysis methods to analyze multimodal data from large populations of neurons. When analyzing large populations of neurons, researchers often use dimensionality reduction techniques to better understand how neural activity of a population changes over time in relation to external factors (Mante et al., 2013; Shenoy et al., 2013; Cunningham and Yu, 2014). With information about additional modalities, we could separately use dimensionality reduction techniques on separate populations of neurons (e.g., those of different cell type; Armañanzas and Ascoli, 2015) to see how they differ. Related to dimensionality reduction techniques, researchers use latent variable models to model shared, but unobservable, variance between neurons (Sahani, 1999; Kulkarni and Paninski, 2007). These models could be better understood with knowledge about other modalities. For example, we could understand whether the shared variability is due to neurons sharing the same morphology, having similar gene expression, sharing synaptic inputs, or sharing neuromodulatory inputs. In general, by simply looking at differences between separate categories of neurons, many of our current analysis techniques can help us understand multimodal data.

Another method for utilizing multimodal data would be to analyze the neural activity as a function of other modalities. In the case of modeling the activity of individual neurons, another modality could act as a covariate in a predictive model. For example, a regression model of spikes could include local field potentials or fMRI as covariates. This would yield insight into how activity at a larger spatial and temporal scale influences local activity, i.e., how more global phenomena affect local, precise activity. As another example, suppose we aim to understand how gene expression is related to neurons’ response properties. First, the response properties (e.g., whether it has phasic or tonic responses to stimuli) could be quantified, and then the expression of many genes could be used as covariates to predict these responses. Predictive models can help us understand how other modalities influence activity of individual neurons.

Similarly, the activity of large populations of neurons can be modeled as a function of other modalities. To do this, the additional modalities can be utilized as constraints in latent variable or dimensionality reduction models. For instance, latent variable models could be enhanced by constraining the latent variables to be consistent with observations from other experimental modalities. That is, only neurons of a specific classification would share latent inputs. Another possibility would be to constrain dimensionality reduction techniques so that different classes of neurons would occupy different dimensions. This approach could be similar to targeted dimensionality reduction (Mante et al., 2013), which uses task-relevant variables as the different dimensions. Essentially, we would want to de-mix the activity into activity caused by each class of neurons. In general, constraints would allow more directly interpreting the outputs of these analysis techniques, to understand how these modalities predict activity.

Lastly, it could be especially beneficial to develop analysis methods that are specifically designed for analyzing multimodal data. Semedo et al. (2014) developed a latent variable model to look at the interaction between separate populations of neurons. While they used their method to investigate interactions between neurons from different brain areas, the technique could be used to look at interactions between any different classifications of neurons (differing morphology, gene expression, etc.). The authors make the important point that an alternative approach would be to first reduce the dimensionality of each population of neurons, and then look at their interaction. However, the separate dimensionality reduction could remove important aspects of the interaction between populations. Thus, their specific method for analyzing both populations simultaneously was important.

In the previous discussion, we have assumed that adding an additional modality would always be beneficial for modeling the neural activity. However, this may not always be the case; there may be explaining away across modalities. For example, if we have a lot of electrophysiological data, then connectomics data may become irrelevant, because the physiology already gives away a lot of connectivity information (Keshri et al., 2013; Fletcher and Rangan, 2014; Veeriah et al., 2015). Similarly, having both connectivity and cell type information may not be very beneficial, because connectivity information can predict cell types (Jonas and Kording, 2015). As such, there are a wide range of scenarios where recording from multiple modalities may not be overly useful. Further multimodal measurements are needed to determine how complementary vs. redundant different data sources are, as we move towards truly large datasets.

In an era when both the amount and the diversity of data is increasing, it’s critical to develop techniques that can help us make sense of this large-scale and multimodal data.

## Author Contributions

JIG and KPK wrote the manuscript. JIG conducted the analyses.

## Funding

We want to thank the NIH for funding (MH103910, NS074044, EY021579, EY025532).

## Conflict of Interest Statement

The authors declare that the research was conducted in the absence of any commercial or financial relationships that could be construed as a potential conflict of interest. The reviewer, PL, and handling Editor declared their shared affiliation, and the handling Editor states that the process nevertheless met the standards of a fair and objective review.

## Footnotes

^1^More details on how we generated Figure 1 are as follows. We tracked how often different modalities of technology were used together over 5 year intervals. We did this by searching the PubMed database for papers with subjects (“MeSH Terms” in the database) representing the modalities. Note that we searched for the terms “genetics” and “gene” for the genetics modality. We find the probability that, out of the neuroscience articles (those appearing when searching for “neuron”, “neural”, or “brain”), the subject matter contains one or two of the modalities. Let *P(M_1_)* be the probability of M_1_ being a subject of a paper, and *P(M_1_, M_2_)* be the probability of both M_1_ and M_2_ being subjects of a paper. The relative co-occurrence (*y*-axis) is *P(M_1_, M_2_)*/*(P(M_1_)* × *P(M_2_))*.

^2^Working on a larger scale than individual neurons (or in the case of *c. elegans*), matching information across subjects is more feasible. For example, to match connectomes, graph matching approaches are being used (Vogelstein et al., 2015). Others have matched information about diseases in humans and animal models using ontologies (Washington et al., 2009; Maynard et al., 2013).
